# Dynamic Three-Dimensional Contrast-Enhanced Ultrasound to Predict Therapeutic Response of Radiofrequency Ablation in Hepatocellular Carcinoma: Preliminary Findings

**DOI:** 10.1155/2018/6469703

**Published:** 2018-08-26

**Authors:** Jiaying Cao, Yi Dong, Feng Mao, Wenping Wang

**Affiliations:** ^1^Department of Ultrasound, Zhongshan Hospital, Fudan University, 200032 Shanghai, China; ^2^Shanghai Institute of Medical Imaging, 200032 Shanghai, China

## Abstract

**Background & Aims:**

To investigate the value of dynamic three-dimensional contrast-enhanced ultrasound (3D-CEUS) in the assessment of therapeutic response of hepatocellular carcinoma (HCC) treated with radiofrequency ablation (RFA).

**Methods:**

Forty-two patients (31 men and 11 women; mean age (52.1 ± 13.1 years)) with 42 clinical diagnosed HCC lesions (size range 14-48 mm; mean size 28.4 ± 9.9 mm) treated by RFA were included. All patients underwent two-dimensional contrast-enhanced ultrasound (2D-CEUS) and 3D-CEUS 1 month after treatment. Two radiologists assessed the absence (complete response, CR) or presence (residual tumor, RT) of any arterially hyperenhancing nodules within or along the margin of the treated HCC lesions. Complete response on magnetic resonance (MR) imaging acted as standard of reference (SOR).

**Results:**

After RFA treatment, 3D-CEUS was successfully conducted in 34 HCC lesions. CR was observed on both 2D-CEUS and 3D-CEUS in 25/42 (59.5%) HCC and RT in 6/42 (14.3%) HCC lesions. In 3/42 (7.1%) HCC lesion, RT was documented by SOR and 3D-CEUS, but it was not appreciable at 2D-CEUS. In 3/42 (7.1%) HCC lesion, the presence of peripheral RT was suspected by both 2D-CEUS and 3D-CEUS, but it was not confirmed by SOR. No statistically significant difference between 2D-CEUS and 3D-CEUS in depicting either CR or RT was found (*P* = 0.25). Combined with dynamic 3D-CEUS, the diagnostic accuracy was improved from 85.7% to 92.9%.

**Conclusions:**

3D-CEUS might be helpful in better diagnostic performance in the assessment of therapeutic response of HCC treated after RFA.

## 1. Introduction

Various nonsurgical local minimal invasive treatment options, including radiofrequency ablation (RFA), transarterial chemoembolization (TACE), and percutaneous alcoholization, have emerged as valid alternative treatments in patients with hepatocellular carcinoma (HCC) [1, 2]. Among which, RFA currently represents the first-line therapy for patients with unresectable HCC [3, 4].

An accurate assessment of therapeutic response is of vital importance for clinical decision-making procedure. Early detection of residual tumor or recurrence area after ablation is key to prompt intervention [5, 6]. With the aim to assess treatment response and to monitor evolution of the ablative tissue over time, clinical, laboratory, and imaging follow-up are usually performed one month after treatment [3, 4]. Medical imaging plays an important role in the evaluation of RFA treatment response of HCC patients. Changes in tumor size and tumor vascularity are the very parameters in evaluation of the therapeutic response. Previously, change in tumor size has been used in clinical practice to determine the treatment response according to the World Health Organization criteria and the Response Evaluation Criteria in Solid Tumors (RECIST) [7]. Since perfusion changes begin earlier than size changes after RFA, the ability to evaluate early perfusion changes of HCC is more crucial [8].

Current imaging modalities mainly focus on tumor size reduction. Dynamic contrast material enhanced computed tomographic (CT) and magnetic resonance (MR) imaging were routinely regarded as the reference standard in the evaluation of tumor response [9]. They were shown to depict treatment induced tumor perfusion changes before there were changes in lesion size [10, 11]. However, their application in clinical assessment of HCC ablation is limited by accessibility of CT and MR imaging equipment, complexity of procedure, relative high cost, risk of allergies, radioactive side effects, contradictions of renal insufficiency, and metal implants.

The application of two-dimensional contrast-enhanced ultrasound (2D-CEUS) in the prediction of tumor response to chemotherapy has been improved to be earlier than using RECIST criteria [12]. However, after injection of ultrasound contrast agents, the brief arterial phase makes it hard to display whole ablative zone or to obtain enough spatial information of residual lesion in a short timeframe. Meanwhile, 2D-CEUS displays tumor perfusion in a single plane, it could not fully display the vascular characteristics if the tumor contains abundant blood vessels or in a position suboptimal detection [13–15].

Recently, dynamic three-dimensional contrast-enhanced ultrasound (3D-CEUS) technique has been reported to improve the display of tumor vascularity, allowing evaluation of the tumor perfusion in three orthogonal planes [16]. Dynamic 3D-CEUS is employed more often in the observation of the spatial morphology of the tumor and its relationship with the surrounding large vessels than in the clinical evaluation of HCC ablation response [17].

The purpose of our current study is to investigate the diagnostic performance of dynamic 3D-CEUS compared with 2D-CEUS in the assessment of therapeutic response of HCC treated with RFA. The influence of 3D-CEUS on clinical outcome was also evaluated.

## 2. Patients and Methods

### 2.1. Patients

Over the periods of July 2016 to October 2017, a total of 42 HCC patients underwent RFA were involved in this study. The target lesion was defined as the only or biggest lesion of each patient. The patients' age ranged from 24 to 71 years (Mean 52.1 ± 13.1 years). The size of target HCC lesions ranged from 14 to 48 mm (Mean 28.4 ± 9.9 mm). The HCC lesions were diagnosed by histological (biopsy or operation) (31 cases), CE-CT (2 cases), and CE-MR imaging (9 cases) results before any treatment. Baseline characteristics of 42 patients were shown in Table 1. All patients underwent RFA treatment after full diagnosis. At 1 month after RFA procedure, CE-MR and CEUS (both 2D-CEUS and 3D-CEUS) were performed to predict the therapeutic response. The patients would be advised to add another procedure of RFA in case that there was a definite evidence of residual tumor on MR images.

Inclusion criteria comprised the following: patients with single lesion of size no more than 50 mm or with up to 3 lesions of size no more than 30 mm; informed consent before ablation; and the quality of 3D-CEUS images meets the requirements of diagnosis.

Exclusion criteria were as follows: patients with contraindications according to the guidelines of the European Federation of Societies for Ultrasound in Medicine and Biology (EFSUMB) [18, 19]. Patients' inability to tolerate CE-MR or dynamic 3D-CEUS examination; and receiving other local tumor therapies between two examinations.

### 2.2. 2D-CEUS

All target lesions were evaluated by 2D-CEUS firstly and then evaluated by dynamic 3D-CEUS. Two experienced radiologists (more than 10 years of CEUS of the liver), who were aware of the patients' clinical histories, performed 2D-CEUS scanning by an Aplio500 ultrasound system (Toshiba, Otawara, Japan), provided with a 3.5 MHz (center-frequency) PVT-375BT convex array probe and contrast harmonic imaging (CHI) software. The ultrasound contrast agent used in the present study was sulfur hexafluoride (SonoVue, Bracco, Milan, Italy), which was injected intravenously as a 2.4 mL bolus followed by 5 mL of normal sterile saline flush using a 20-Gauge peripheral intravenous cannula. A very low mechanical index (MI), ranging from 0.05 to 0.08, was used for real-time imaging.

### 2.3. 3D-CEUS

After carefully determined the position and orientation of the probe contacted after 2D-CEUS, 3D-CEUS was performed in the same session with an interval time of at least 15 min. Dynamic 3D-CEUS was performed by the same ultrasound unit provided with a volumetric mechanical 3.5 MHz (central frequency) PVT-382 MV convex array probe and CHI software. SonoVue was then injected intravenously as a 2.4 mL bolus followed by 5 mL of normal sterile saline flush. The “4D” and “contrast” scanning mode were started in succession when the lesion was displayed definitely and clearly. The imaging window and transducer orientation were optimized to minimize out-of-plane motion. MI was set in the range of 0.10-0.14. Raw data of more than 3 min was stored for postprocedure analysis and the major observation period was focused on the first 1 min. During dynamic 3D-CEUS acquisition process, the probe was kept stable in the scanning area. Patients were instructed to keep quiet breathing during imaging process to reduce interference of great respiratory motion.

### 2.4. Imaging Analysis

Volumes of 3D-CEUS were stored in raw data format and reviewed using the built-in proprietary software (CHI-Q, Version 5.01, Toshiba, Japan). Dynamic 3D-CEUS images can be postprocessed by rotation, adjustment of the contrast ratio, etc. The “multislice” display mode allows multisection observation from arbitrary orientation of the 3D dynamic images. The function of “inversion” can be used to investigate the shape of perfusion defect area. Two experienced readers were also asked to report (both 2D-CEUS and dynamic 3D-CEUS) independently: (1) complete response (CR): the absence of any nodular arterially enhancing portion within or at the margin of treated HCC; (2) residual tumor (RT): any nodular arterially hyperenhanced area within or along the edge of a treated HCC indicated incomplete ablation [16, 20–22]. The presence of a uniform and thin (4-5 to 7-8 mm thick) peripheral rim of contrast enhancement surrounding the treated zone was regarded as benign reactive hyperemia on CEUS [20], while if there existed divergence on either 2D-CEUS or 3D-CEUS dynamic images, the images would be discussed until agreement was reached.

Clinical assessment and laboratory data were also applied to confirm the diagnosis in case the imaging results contradicted one another. As to RT, dynamic 3D-CEUS can obtain the residual area on the 3 orthogonal planes at peak enhancement and the ratio of the volume of residual area to the whole lesion can also be calculated.

#### 2.4.1. Reference Standard-MR Imaging (SOR)

MR images are considered the most accurate modality for tumor response assessment following liver thermal ablation and early detection of RT. Enhanced MR imaging was performed on 1.5T clinical MR imaging unit (Magnetom Aera; Siemens, Erlangen, Germany) during 3 days before and after CEUS. MR contrast agent, gadopentetate dimeglumine (Magnevist, Bayer Schering Pharma), was injected intravenously. The area, which presented typical hyperperfusion in the arterial phase within or along the margin, was regarded as RT. CR was considered without any enhancement within or at the margin of the ablative zone.

After RFA treatment, CE-MR scans were performed at an interval no more than 3 days of 2D-CEUS and 3D-CEUS. Diagnostic criteria for complete treatment at MR imaging were the absence of any enhancing portion within or at the margin of the ablative zone during the hepatic arterial phase, as previously reported [23]. Diagnostic criteria for residual viable tumoral tissue at CE-MR imaging were any nodular arterially enhancing area within or along the margin of the treated HCC [21].

### 2.5. Statistical Analysis

On the basis of SOR findings, detailed lesion-by-lesion analysis was performed and sensitivity and specificity (diagnostic performance) for 3D-CEUS were calculated. The statistical significance of the difference between 2D-CEUS and 3D-CEUS in the assessment of RFA therapeutic response of HCC was tested by two-tailed McNemar's test with the continuity correction. Kappa value was used to check their consistency. The diagnostic performance, including sensitivity, specificity, positive predictive value (PPV), negative predictive value (NPV) and accuracy with 95% CI for 2D-CEUS, and the combination of 2D-CEUS and 3D-CEUS, was calculated by Fisher's exact test. Statistical significance was considered to be present at a* P* < 0.05.

### 2.6. Institutional Board Approval

This prospective study was approved by the institutional review board of our institution. Informed consent was waived before 3D-CEUS examination. The procedure followed was in accordance with the Declaration of Helsinki.

## 3. Results

No adverse events have been registered in our patients during or immediately after injection of contrast agent.

Dynamic 3D-CEUS of the ablative zone was acquired in all cases. Among which, the valid adoption rate of 3D-CEUS was achieved in 34 HCC lesions (81.0%, 34/42). Eight HCC lesions displayed not clear enough 3D-CEUS images and, as a result, their dynamic 3D data were not used to assess the therapeutic response. The reasons why they had poor display quality were as follows: 3 lesions were located along with the diaphragm, 3 lesions were at a depth about 100-120 mm from the probe surface, and 2 patients breathed too rapidly. During the brief arterial phase, 3D-CEUS failed to capture the enhancement of these lesions as a whole. A poor display contributed to lack of confidence in the judgment of RT.

### 3.1. Evaluation of Complete Response

**Figure 1:**
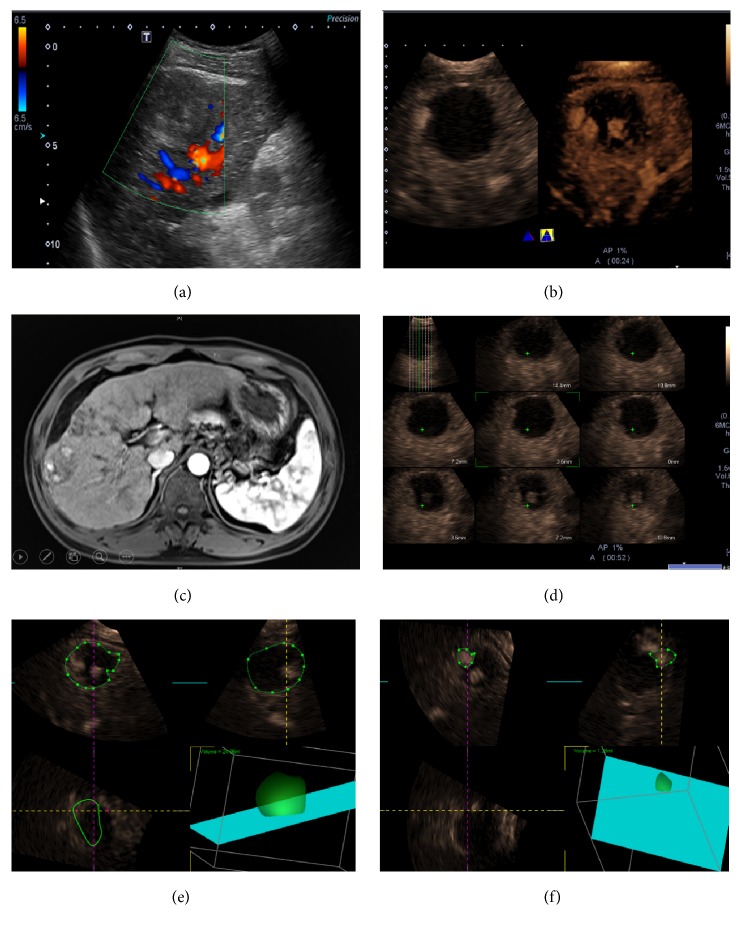
Residual tumor after radiofrequency ablation (RFA). Conventional ultrasound image in a 65-year-old man showed a 45 mm sized homogeneous hypoechoic lesion in the right hepatic lobe. No color signal can be detected inside the lesion by color Doppler flow imaging (CDFI) (a). There was a small enhanced area along the border of the lesion on dynamic 3D-CEUS (right) other than 2D-CEUS (left) images (b). Contrast MR imaging showed confirmed RT inside the lesion with local enhancement (c). The “multislice” display mode showed the RT area on several slices of 3D-CEUS image (d). The contour of the whole lesion was depicted manually and its corresponding volume was calculated automatically (e). The volume of the 3 nodular RT area can also be calculated. The residual proportion added up to 13.4% (f).

**Figure 2:**
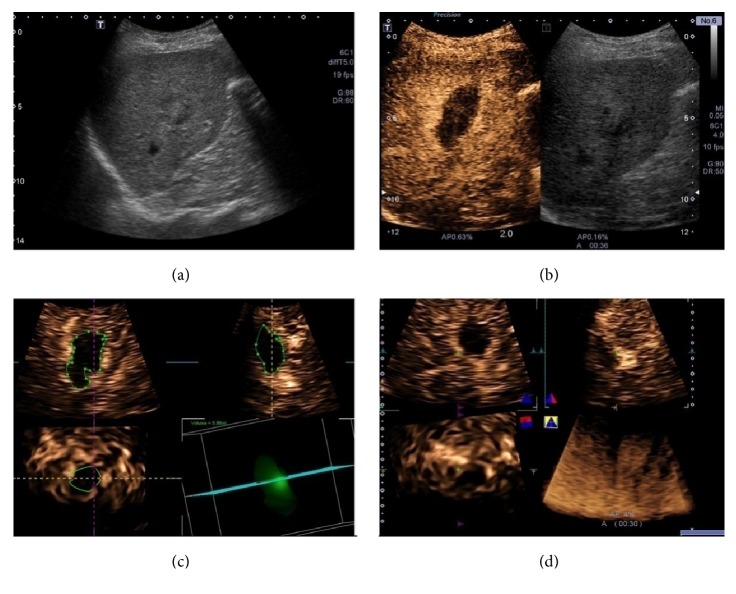
Complete response after RFA. B mode ultrasound image in a 65-year-old man showed a 25 mm sized inhomogeneous hypoechoic lesion in the right hepatic lobe (a). 2D-CEUS showed the nonenhanced necrotic area was larger than the target lesion (b). The contour of the ablative zone was depicted manually and its volume was 5.88ml (c). “Inversion” mode showed the nonenhanced area on 3D-CEUS image (d).

By SOR, 78.6% (33/42) HCC lesions after RFA (size range: 14-45 mm; mean size 27.1 ± 10.2 mm) were confirmed as CR, while 21.4% (9/42) lesions (size range: 18-48 mm; mean size 32.0 ± 9.9 mm) were documented as RT (Figure 1). CR was observed on both 2D-CEUS and 3D-CEUS in 25/42 (59.5%) HCC (Figure 2) and RT in 6/42 (14.3%) HCC lesions. In 5/42 (11.9%) HCC lesions, CR was observed on 2D-CEUS but was not definitely determined on 3D-CEUS.

### 3.2. Detection and Contrast Enhancement of RT Areas

An arterial nodular hyperenhanced pattern was observed in 3 HCC lesions (3/42, 7.1%) both on 2D-CEUS and on 3D-CEUS; 3 HCC lesions were (3/42, 7.1%) only on 3D-CEUS while not on 2D-CEUS images; and 3 HCC lesions were (3/42, 7.1%) only on 2D-CEUS images while not successfully displayed on 3D-CEUS images. All the 9 lesions (9/42, 21.4%) were regarded as RT proven by SOR and treated immediately.

A thin rim enhanced pattern was observed in 7 HCC lesions both on 2D-CEUS and on 3D-CEUS images, whereas a rough rim pattern was observed in 3 HCC lesions. The former was regarded as CR and the latter suspected RT by CEUS; however, all the 10 lesions were diagnosed as CR by SOR for perfusion defects on MR images (Table 2).

The enhanced volume and the whole lesion volume of the RT lesion determined by 3D-CEUS were contoured on the plane, and the ratio of enhanced area to the whole lesion was calculated. It showed that residual proportion was 10.0%  ±  4.5% (range: 4.6%-15.9%) (Figure 1).

### 3.3. 2D-CEUS vs 3D-CEUS

**Figure 3:**
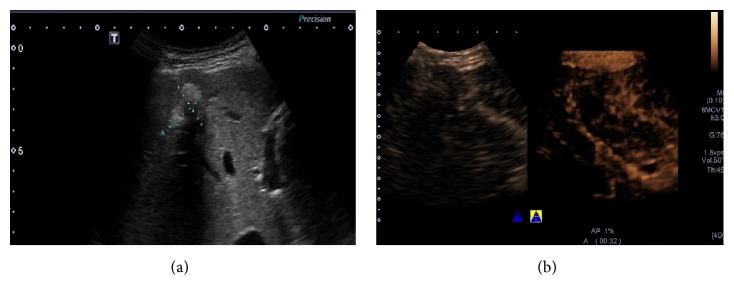
Presence of attenuation due to RFA treatment. B mode ultrasound image in a 71-year-old man showed a 25 mm sized lesion treated by RFA in the right lobe of the liver. It had posterior attenuation caused by scars or necrosis (a). Its posterior border cannot be demonstrated by 2D-CEUS (left), whereas it can be shown on dynamic 3D-CEUS. The relationship of the lesion and the posterior adjacent large vessels can also be shown (b).

The posterior border of the lesions (11.9%, 5/42) cannot be demonstrated on 2D-CEUS in the case of presence of attenuation due to RFA treatment, whereas it can be replenished on dynamic 3D-CEUS (Figure 3). Due to the particular advantage of 3D-CEUS, a nodular RT area was observed in 1 lesion on 3D-CEUS while not on 2D-CEUS images in our study.

For 34 HCC lesions evaluated by both 2D-CEUS and 3D-CEUS, 3D-CEUS and 2D-CEUS provided the same diagnosis in 91.2% (31/34) cases, showing excellent intermodality agreement. There was not any statistically significant difference between conventional 2D-CEUS and 3D-CEUS in depicting either CR or RT (*P* = 0.25). They had a good consistency (*κ* = 0.75).

### 3.4. Diagnostic Efficacy of 2D-CEUS Combined with 3D-CEUS

In 3/42 (7.1%) HCC, RT was documented by SOR and 3D-CEUS, but it was not appreciable at 2D-CEUS. In 3/42 (7.1%) HCC, the presence of peripheral RT was suspected by both 2D-CEUS and 3D-CEUS, but it was not confirmed by SOR.

On a lesion-by-lesion basis, sensitivity, specificity, positive and negative predictive values, and accuracy of 2D-CEUS were 66.7% (95%CI 0.299-0.925), 90.9% (95%CI 0.757-0.981), 66.7% (95%CI 0.299-0.925), 90.9% (95%CI 0.756-0.981), and 85.7% respectively. The corresponding indexes of the combination of 2D-CEUS and 3D-CEUS were 100% (95%CI 0.541-1.000), 91.7% (95%CI 0.775-0.983), 66.7% (95%CI 0.299-0.925), 100% (95%CI 0.894-1.000), and 92.9% (Table 3).

## 4. Discussion

Due to recent advances in RFA and its use for therapeutic indications, the commonly used RECIST [3, 4, 24] criteria based on the size of HCC lesions do not fulfil the requirements for functional assessment of tumor response, and new imaging modalities are needed [25].

### 4.1. Comparison between 2D-CEUS and 3D-CEUS

The changes of the vascularization of HCC could be interpreted as a response to a certain therapy [26]. According to the current EFSUMB guidelines, dynamic CEUS examinations should ideally be reproducible irrespective of the ultrasound equipment, data acquisition, and analysis software used [18, 19]. The advantages of CEUS include a good safety profile, simplicity, patient tolerance, lack of ionizing radiation, and real-time multiplanar imaging capability [18, 19]. Previously, in the case of percutaneous therapies of malignant liver tumors, CEUS enables the assessment of tumor microvascularization (particularly arterial hypervascularization) between treatments [27]. This may contribute to an improved therapeutic monitoring in locoregional treatments [28]. Other results illustrated the dependence of 2D-CEUS imaging on operator' accuracy because the operator has to find the exact same 2D plane to accurately monitor longitudinal perfusion changes, which is not possible in clinical practice [29–31].

As shown in our result, 3D-CEUS can reduce false positive rate, as dynamic 3D-CEUS images have the advantages of showing enhanced images in 3D plane and providing more detailed spatial information compared with the 2D-CEUS images. In comparison to conventional 2D-CEUS, 3D-CEUS could objectively depict tumor vascularity and intratumoral perfusion by reconstructing stereoscopic images [32]. The capability of clearly display the posterior information of the target lesions with posterior attenuation is another remarkable advantage of dynamic 3D-CEUS, as shown in Figure 3. Luo et al. found that 3D-CEUS was useful in the evaluation and characterization of vascular patterns of focal liver tumors [33]. Xu et al. described the advantages of 3D-CEUS in the evaluation of 51 lesions in 51 patients with liver cancer who underwent local therapies and found that 3D-CEUS not only enhanced the diagnostic confidence in the majority of the patients but also changed the management of some patients [34]. Depending on expertise of operators, not all RT areas can be observed on 2D-CEUS images in such a short arterial phase and 3D-CEUS can make up for the deficiency to some extent, especially for nodular enhanced RT, as shown in Figure 1. Combined with dynamic 3D-CEUS, CEUS produced satisfactory results for the evaluation of treatment response in HCC patients after RFA therapies. 3D-CEUS imaging could more accurately measure tumor perfusion for the entire target lesion, which is critically needed for long-term monitoring of treatment response in HCCs.

### 4.2. 3D-CEUS Enhancement Pattern of Residual HCC Lesion after RFA

Dynamic 3D-CEUS is equipped with new kinetic imaging capability and can continuously display dynamic perfusion process in the tumor depend on postacquisition reconstruction. This modality is particularly valuable for illustrating the tumor enhancement kinetics during the arterial phase. In our study, most of residual HCC lesions after RFA appeared as irregular hyperenhanced nodules of the lesions during the arterial phase, which also showed hypoenhancement during the portal venous and late phase on dynamic 3D-CEUS images. The stereoscopic visualization of residual HCC morphology is most obvious during arterial phase. However, the false positive rate will occur in case the rough rim enhancement would be mistaken as the overlap of inflammatory zone and nodular residual HCC on both 2D-CEUS and 3D-CEUS. Rough rim enhanced pattern is quite equivocal and, as a matter, the 3 lesions that showed this pattern were classified CR at SOR. The result suggests that the rim enhancement pattern might be a source of misdiagnosis and its clinical significance should be questioned.

Dynamic 3D-CEUS is superior to 2D-CEUS in displaying the spatial relationship of HCCs and their vascularity patterns and simultaneous imaging of HCC perfusion and anatomic features [35]. Dynamic 3D-CEUS can be shown in “multislice” displaying mode, which can help display RT alongside the border of the HCC lesions. It can display the size and shape of RT by depicting its enhanced contour and obtain the ratio of RT to the whole lesion in order to guide further treatment. It also allows observation on the ROI from different perspective as well as volume reconstruction to display stereoscopic morphology of the structure of interest. Dynamic 3D-CEUS has revealed more details of the boundary and RT area of HCC.

## 5. Limitation

The obvious limitation of dynamic 3D-CEUS is that the lesions at remote location are not suitable to be evaluated. It is difficult for 3D-CEUS to capture the enhancement of RT in such a relative short arterial phase in the case the lesion is in low displaying quality. However, 2D-CEUS has the advantage in displaying the lesions located in deep or marginal area. In view of this, dynamic 3D-CEUS is currently unable to replace the role of 2D-CEUS in therapeutic response evaluation of HCC. A current limitation was found to be the lack of TIC and quantitative indexes analysis, which might be more sensitive in evaluating early treatment response. More studies are needed to evaluate whether dynamic 3D-CEUS quantitative analysis could monitor RFA therapy in HCC lesions. Another major limitation of CEUS imaging is the respiratory motion. As proposed in the current study, keeping quiet breathing during imaging process is probably a good alternative.

## 6. Conclusion

It is feasible to use dynamic 3D-CEUS as an assistance to 2D-CEUS to evaluate early response and monitor therapy response of RFA in HCC patients. The combination of 2D-CEUS and 3D-CEUS might have potential to be applied as an alternative to CE-MR imaging in clinical follow-up assessment after RFA treatment in HCC patients.

## Figures and Tables

**Table 1:** Baseline characteristics of 42 patients with 42 HCC lesions in our study.

Characteristic	Value (n = 42 patients)
Male/female	31/11
Age (y)	
Mean ± standard deviation	52.1 ± 13.1
Range	24 - 71
Diagnosis	
Histological result	31
CE-CT	2
CE-MR	9
Number of HCC lesions	
Solitary	28
Multiple	14
Size of the target lesions (mm)	
Mean ± standard deviation	28.4 ± 9.9
Range	14 - 48
Liver disease	
Liver cirrhosis	38
Chronic liver disease	4

**Table 2:** Contrast appearances of 42 HCC lesions observed by two modalities of CEUS and the corresponding SOR results.

Contrast enhancement pattern	Number of lesions
**RT confirmed by SOR**	**9**
Nodular enhance pattern(RT)	
Observed on 2D-CEUS alone (not successfully displayed on 3D-CEUS)	3
Observed on 3D-CEUS alone (not observed on 2D-CEUS)	3
Observed on both 2D-CEUS and 3D-CEUS	3

**CR confirmed by SOR**	**33**
Rim enhanced pattern (CR)	
Thin (observed on both 2D-CEUS and 3D-CEUS)	7
Rough (observed on both 2D-CEUS and 3D-CEUS)	3
Without enhancement(CR)	
Observed on both 2D-CEUS and 3D-CEUS	18
Observed on 2D-CEUS alone (not definite determined on 3D-CEUS)	5

CEUS: contrast-enhanced ultrasound; SOR: standard of reference; RT: residual tumor; CR: complete response.

**Table 3:** Comparison of the diagnostic efficacy of 2D-CEUS and 2D-CEUS combined with 3D-CEUS.

Diagnostic performance	2D-CEUS	2D-CEUS + 3D-CEUS
Sensitivity	6/9 (66.7 %)	6/6 (100 %)
Specificity	30/33 (90.9 %)	33/36 (91.7 %)
PPV	6/9 (66.7 %)	6/9 (66.7 %)
NPV	30/33 (90.9 %)	33/33 (100 %)
Accuracy	36/42 (85.7 %)	39/42(92.9 %)

2D-CEUS: two-dimensional contrast-enhanced ultrasound; 3D-CEUS: three-dimensional contrast-enhanced ultrasound; PPV: positive predictive value; NPV: negative predictive value.
